# TAT-Mediated Acidic Fibroblast Growth Factor Delivery to the Dermis Improves Wound Healing of Deep Skin Tissue in Rat

**DOI:** 10.1371/journal.pone.0135291

**Published:** 2015-08-13

**Authors:** Long Zheng, Qi Hui, Lu Tang, Lulu Zheng, Zi Jin, Bingjie Yu, Zhitao Wang, Peng Lin, Weidan Yu, Haiyan Li, Xiaokun Li, Xiaojie Wang

**Affiliations:** 1 School of Pharmacy, Wenzhou Medical University, Chashan University Park, Wenzhou 325035, China; 2 Ministry of Education Engineering Research Center of Bioreactor and Pharmaceutical Development, Jilin Agricultural University, Changchun, 130118, China; Northwestern University, UNITED STATES

## Abstract

**Background:**

The definition of deep tissue injury was derived from multiple clinical cases as “A purple or maroon localized area of discolored intact skin or blood-filled blister due to damage of underlying soft tissue from pressure and/or shear”. Acidic fibroblast growth factor (aFGF) significantly improves wound healing under diabetic conditions. However, to date, the therapeutic application of aFGF has been limited, due to its low delivery efficiency and short half-life.

**Methodology/Principal Findings:**

Using an animal model of magnet-induced pressure ulcers, transactivator of transcription protein (TAT)-aFGF was evaluated for transdermal delivery and wound healing. Immunohistochemistry and Western blotting were also performed to determine the expression of transforming growth factor (TGF)-β1, α-smooth muscle actin (α-SMA), CD68, proliferating cell nuclear antigen (PCNA) and TGF-β-receptor II (TGF- βRII) in cultured human dermal fibroblasts. We found that that mice treated with TAT-aFGF had higher accumulation of aFGF in both dermis and subcutaneous tissues compared with mice treated with aFGF alone. In the remodeling phase, TAT-aFGF treatment decreased the expression of α-SMA to normal levels, thereby facilitating normal wound healing processes and abrogating hypertrophic scarring. In human dermal fibroblasts, TAT-aFGF reversed the suppressive effect of TNF-α on α-SMA expression and restored TGF-βRII and TGF-β1 expression.

**Conclusions/Significance:**

Our results demonstrate that TAT-aFGF has a favorable therapeutic effect on the healing of subcutaneous deep tissue injury.

## Introduction

In 2007, deep tissue injury (DTI) was first described by the National Pressure Ulcer Advisory Panel as the newest type of pressure ulcer in the updated staging system [1]. Derived from multiple clinical cases, the definition of DTI was described as ‘A purple or maroon localized area of discolored intact skin or blood-filled blister due to damage of the underlying soft tissue from pressure and/or shear’ [1]. Pressure-related DTI under intact skin in humans may result from a single event of prolonged immobilization, such as a lengthy surgical operation. Studies in animal models have shown that pressure-related ischemia of both subcutaneous tissue and muscle can occur under intact skin [2]; however, there are currently no specific treatments recommended [3].

The fibroblast growth factor (FGF) family regulates developmental processes and tissue homeostasis, including brain patterning, vascular branching morphogenesis and limb development [4]. Fibroblasts are the major mesenchymal cell type in connective tissue and deposit the collagen and elastic fibers of the extracellular matrix (ECM) [5]. Although multiple growth factors, including epidermal growth factor, platelet-derived growth factor, and vascular endothelial growth factor, participate in tissue reconstruction, acidic FGF (aFGF) plays a pivotal role in regulating fibroblasts [6,7], which are central to wound healing in DTI. Previous studies have reported that the administration of aFGF significantly improves wound healing under diabetic conditions [8]. It has been shown that aFGF also enhances local generation of tissue collagen and increases levels of transforming growth factor (TGF)-β1 and proliferating cell nuclear antigen (PCNA) which appear to be involved in the mechanisms underlying wound healing [9]. However, as for many therapeutic proteins, the pharmacological action of aFGF is limited because of low local delivery efficiency. Drug delivery systems such as protein transduction domains, nanoparticles and liposomes have therefore been exploited for the improvement of therapeutic delivery of protein drugs [10,11].

Transactivator of transcription protein (TAT) was discovered by the Frankel and Pabo [12] and Green and Loewenstein [13] groups independently in 1988. It contains a so-called cell-penetrating peptide that mediates the translocation of biological agents from membrane barriers into live cells. This makes TAT a potential vehicle for drug delivery, although the mechanism of its accumulation in cytoplasm is not fully understood [14,15]. The fusion of TAT to metallothionein was shown to enhance metallothionein delivery, thereby inhibiting cell apoptosis, reducing fibrosis and restoring cardiac function in a myocardial ischemia/reperfusion model [16]. Moreover, our previous study demonstrated a potential role for TAT in the delivery of human aFGF_19-154_ from the surface of the eyeball to the retina in rats [17]. The purpose of this study was to investigate the efficiency of TAT-mediated aFGF delivery in dermal and subcutaneous tissues and evaluate its effectiveness for treating DTI beneath intact skin.

## Materials and Methods

### Delivery gel

Our previous studies showed that the TAT-aFGF fusion protein is stable *in vitro* [18] and that TAT does not affect the bioactivity of aFGF *in vivo* [17]. The delivery gel containing aFGF or TAT-aFGF was prepared as below. Briefly, 0.25g Carbopol (Sigma-Aldrich, St. Louis, MO) was added to deionized water (45 ml) containing 0.5 ml glycerol (Sigma-Aldrich) and allowed to swell overnight. Methylparaben (0.25 g) and ethylparaben (5 mg) (Sigma-Aldrich) were mixed in 1 ml of phosphate buffered saline (PBS). The pH was immediately adjusted to 7.0 with triethanolamine solution (Sigma-Aldrich). The gel was sterilized for 20 min at 121°C. After the solution was cooled to room temperature, 3 ml of protein solution (15 mg TAT-aFGF or aFGF with 0.5 g of serum albumin) was added to the gel. The gel was then dispensed into aluminum tubes, sealed and stored at 4°C.

### Animals

48 male Sprague-Dawley rats (300–350 g) and 48 male BALB/c mice (18–22 g) (Silaike, Shanghai, China) were maintained in a specific pathogen-free (SPF) animal facility which had controlled temperature and humidity and a 12-hour dark/light cycle. The animals were allowed free access to standard laboratory food and water. All animal protocols were approved by the Institutional Animal Care and Use Committee (IACUC) of Wenzhou Medical University.

### Transdermal delivery of TAT-aFGF proteins in mice

BALB/c mice were anesthetized with chloral hydrate (300 mg/kg, Sigma-Aldrich) and their dorsal hair (3cm×3cm) was carefully shaved. Around 24 h later, 50 μl of aFGF or TAT-aFGF solution (60 μg/ml) was topically administrated to the shaved dorsal skin. Blank gel was used as a control solution. At different time points (0, 30 min, 2 h and 8 h), mice were euthanized by cervical dislocation. The skin tissues were harvested and then fixed in 4% paraformaldehyde before embedding for paraffin sections.

### Rat model of pressure ulcers

The model of pressure ulcers was constructed as described previously, but with some modifications [19]. The greater trochanters of the rats were extensively shaved and subsequently pinched between two ceramic disc magnets which were 8 mm in diameter, 4 mm in thickness, 2.4 g in weight and 3,500G in strength. The cycles of ischemia–reperfusion injury were then performed. A single cycle consisted of a dorsal skin magnet pinch for 12 h followed by a rest period of 12 h. This procedure was done consecutively for two days. A single wound with identical size of 0.5 cm in diameter was created in each rat. After the wounds were established, 200 μl of aFGF, TAT-aFGF solution (300 μg/ml) or blank gel was administrated topically. The treatment was repeated daily for 14 days. The ulcers were monitored using digital images which were used to calculate the wound areas using Image Software. Rats were anesthetized with chloral hydrate (300 mg/kg, Sigma-Aldrich) at 0, 3, 7 and 14 days post-treatment. At different time points, the wound contraction was measured quantitatively [20].

### Histopathological evaluation

At time of sacrifice, tissues of skin ulcers (1.5 cm ×1.5 cm) were harvested from rats, followed by fixation in 4% paraformaldehyde and embedding in paraffin. Conventionally, sections were stained with haematoxylin and eosin (HE) and Masson (Sigma-Aldrich). The penetrating effect of TAT-aFGF on the injured skin was evaluated by immunohistochemistry, in which the primary antibodies for aFGF, TGF-β1, α-smooth muscle actin (α-SMA), CD68 or PCNA (Santa Cruz, CA) were used accordingly. The CD68 and PCNA levels in each group were quantified using Image-Pro Plus software (Nikon, Tokyo, Japan). The positivity density of α-SMA and TGF-β1 was scored semi-quantitatively as 1 (absent), 2 (low), 3 (medium), 4 (strong), and 5 (very strong) by two observers who were blinded to the grouping [21,22]. Similarly, the HE and Masson-stained sections were semi-quantitatively scored at a range of 0 to 4 according to the level of collagen enrichment.

### Apoptotic DNA fragmentation analysis

The apoptosis ratios of injured tissues were assessed by DNA terminal dUTP nick-end labeling (TUNEL) (Roche, Mannheim, Germany) according to the manufacturer’s instructions. The omission of terminal deoxynucleotidyl transferase in tissue sections was used as negative control. The TUNEL index (multiplied by 100) was determined by the ratio of TUNEL-positive nuclei to the total number of nuclei in three random fields under the light microscope.

### Cell culture

The human dermal fibroblast cell line (bought from ATCC, cell line number is PCS-201-012) was a gift from the Institute of Molecular Pharmacology of Wenzhou Medical University. As described previously [23], cells were cultured in Dulbecco’s Modified Eagle’s Medium (DMEM) (low glucose) containing 10% fetal bovine serum (FBS) (Gibco, CA) with 0.1% antibiotics. A suspension of 300,000 cells was seeded onto 6-well tissue culture plates. TNF-α (5 ng/ml), TAT-aFGF (10 or 100 ng/ml) or the combination of TNF-α and TAT-aFGF were added into culture media supplemented with 0.1% FBS. Similarly, aFGF was used as a control in cell cultures.

### Western blot analysis

Western blotting was performed as described previously, with some modifications [24]. The α-SMA, TGF-β1 and TGF-βRII proteins were detected in whole lysates of human dermal fibroblasts. Equal amounts of cell lysate protein (70 μg) were separated on 12% SDS-PAGE gels and Western blotting was subsequently performed. The blots were probed with primary antibodies against α-SMA, TGF-β1 or TGF-βRII. Horseradish peroxidase-conjugated secondary antibodies were used accordingly. The membranes were stripped and re-probed with GAPDH antibody as a protein-loading control. Finally, the relative protein levels were quantified by Bio-Rad software.

### Statistical analysis

All data are presented as mean ± standard error of the mean (SEM). A statistical analysis of the difference between triplicate sets of experiments was performed using a Student’s t-test in GraphPad Prism Software, assuming a double-sided independent variance with *P* < 0.05 considered significant.

## Results

### TAT promotes aFGF penetration in skins

**Fig 1:**
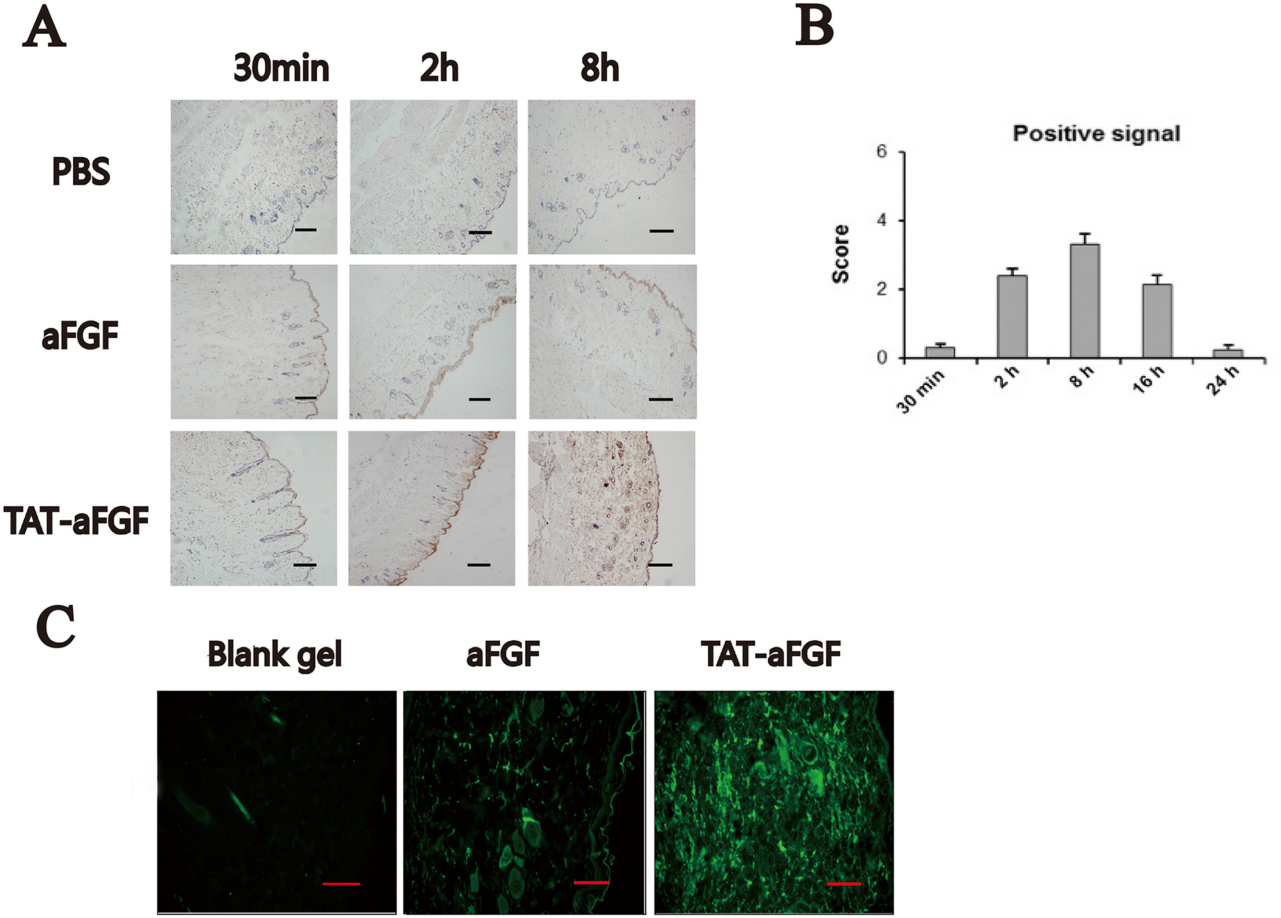
Effect of TAT on aFGF penetration after topical administration in skin. **A:** Under anesthesia, BALB/c mice received topical administration of PBS, aFGF, or TAT-aFGF solutions. Skin tissues were then harvested at the indicated time points. A positive signal of aFGF was detected at 2 h and 8 h in the TAT-aFGF group. Bar = 100 μm. **B:** The positive signal for aFGF was scored semi-quantitatively (0 = absent, 1 = low, 2 = medium, 3 = strong, 4 = very strong) and showed gradual reduction of signal in the aFGF group. **C:** TAT-aFGF aFGF or blank gel were applied at the end of second loading. Tissues were harvested 5 h later and then processed routinely for embedding. Immunofluoresence was performed to measure aFGF accumulation (green) in the dermal and subcutaneous tissues. In the control and aFGF groups, few cells were stained, except some in corneous and hair follicles, while in the TAT-aFGF group, strongly positive staining was detected under the epidermis. Bar = 100 μm.

Previously, we demonstrated that TAT-conjugated aFGF-His6 (TAT-aFGF-His6) exhibited an efficient penetration into the retina following topical administration to the ocular surface [17]. To further understand its transdermal potential, we topically applied TAT-aFGF to the surface of dorsal skin of BALB/c mice. The results demonstrated positive signals of TAT-aFGF in the skin of TAT-aFGF group from 2 h to 8 h after administration (Fig 1A). Primarily, TAT-aFGF accumulated in the hair follicles and subcutaneous tissues. These positive signals decreased gradually from 8 h after administration and were undetectable after 24 h (Fig 1B). In contrast, mice treated with aFGF showed significantly weaker epidermal uptake of aFGF compared with the TAT-aFGF group, with absorption levels similar to that of the control group (Fig 1A).

To assess the TAT mediation of aFGF accumulation in dermal and subcutaneous tissues, we applied TAT-aFGF, aFGF or blank gels in the rat ulcer model. The results showed that aFGF alone weakly crossed the epidermis and accumulated in hair follicles, whereas higher levels of fluorescence were detected in or around cell nuclei in the epidermis from TAT-aFGF treated mice (Fig 1C).

### TAT-aFGF enhances wound healing in rats

**Fig 2:**
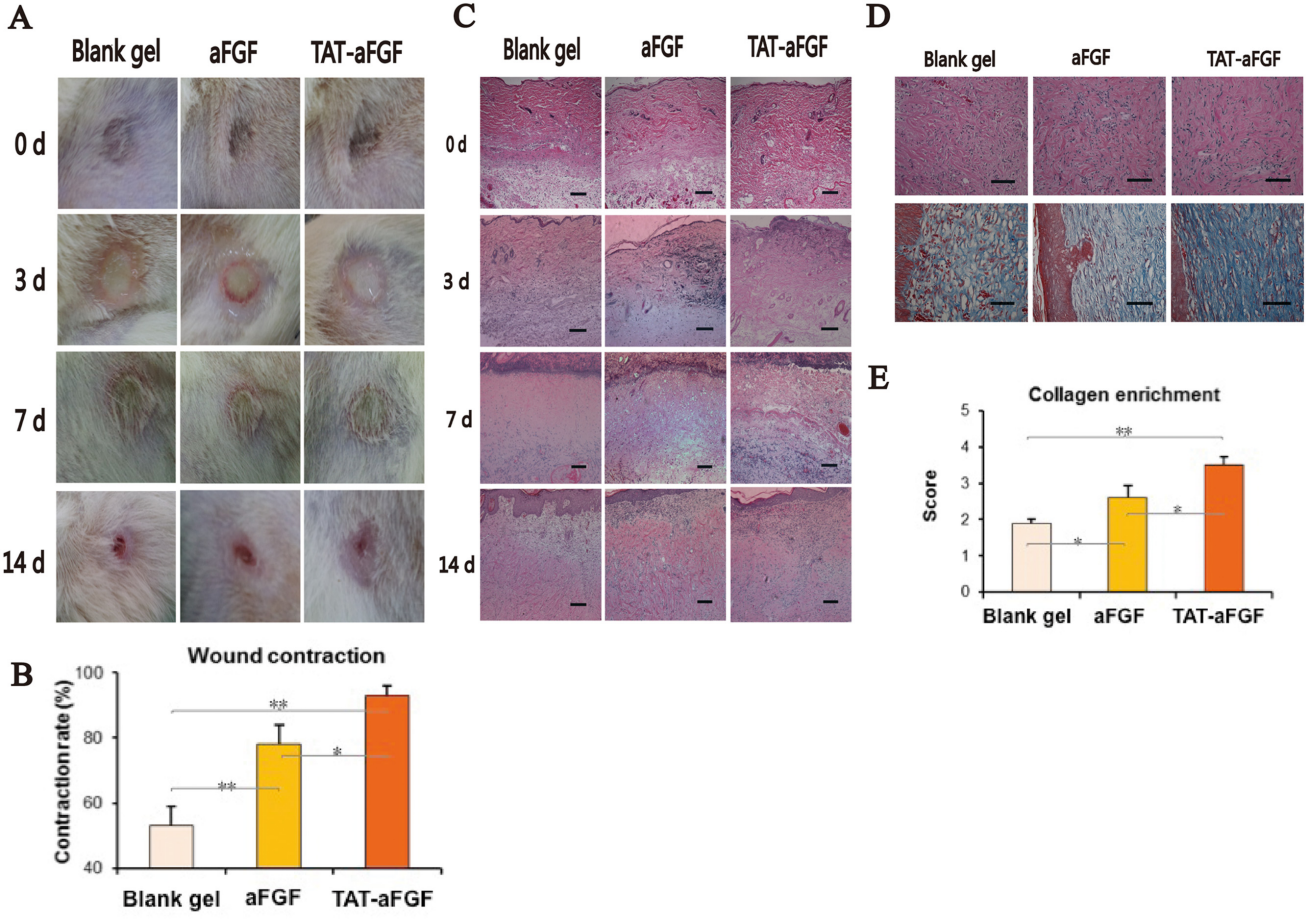
Evaluation of pressure-induced deep tissue injury. **A:** Typical macroscopic views of the TAT-aFGF, aFGF and control groups on day 0, 3, 7, and 14. The red open ulcers remained differentially in the three groups on day 14. **B:** Wound contraction was measured quantitatively, showing a higher rate in the TAT-aFGF group on day 14; * *p* < 0.05, ** *p* < 0.01. **C:** Typical microscopic views (×100) of hematoxylin and eosin (HE) staining of each group on days 0, 3, 7 and 14. Edema and swelling were obvious on day 0. Bar = 100 μm. **D:** HE staining and Masson staining of tissue biopsies on day 14. Bar = 100 μm. **E:** Semi-quantitative scoring of sections from mice on day 14. * *p* < 0.05, ** *p* < 0.01.

Pressure loading on the skin has been used to induce deep ulcers [25]. In our study, on day 1 or 3 after pressure-loading in rats, no open ulcer had formed in the skin. However, the injured skin gradually became yellow and hard. On day 10 or 11, the necrotic skins separated from the epidermis and the red open ulcers subsequently formed. During the healing phase, the skin ulcer gradually decreased in size and disappeared from day 14 after treatment (Fig 2A). There was no visible difference between the three groups before the necrotic skins separated from the epidermis. Moreover, on day 14, the skin ulcers with TAT-aFGF treatment had almost closed. In contrast, the control and aFGF groups still had open wounds (Fig 2A). The wound contraction rate of TAT-aFGF was higher than the other two groups (Fig 2B).

The skin ulcers were analyzed histologically, showing that on day 0, there was swelling and necrotic fat cells, blebbing of the vascular wall and early necrosis of follicular units (Fig 2C). On day 3, although the edema in skins and subcutaneous tissue had decreased, necrosis appeared in the epidermis and follicular units. Prominent polymorphonuclear infiltration and necrosis was found throughout the dermis and subcutaneous tissues (Fig 2C). On day 7, eschars started to form and the skin tissues started to undergo regeneration. During hypertrophic scar formation, the myofibroblasts continued to remodel the ECM that induces connective-tissue contracture [26]. On day 14, this effect was more pronounced in the control groups than in the TAT-aFGF group (Fig 2C), suggesting that TAT-aFGF inhibits connective-tissue contracture by enhancing recovery capacity while reducing recovery duration.

Masson staining in the three groups on day 14 showed that all wounds were filled with the newly formed collagen-enriched ECM (Fig 2D). Wounds in the control and aFGF groups showed deficient collagen deposition and immature tissue organization, whereas a more compact and organized dermis with an abundance of collagen bundles was observed in the TAT-aFGF group (Fig 2D). Semi-quantitative scoring showed more collagen enrichment in the TAT-aFGF group when compared with the other two groups (Fig 2E).

### TAT-aFGF transdermal delivery in rats reduces cell apoptosis but enhances proliferation

**Fig 3:**
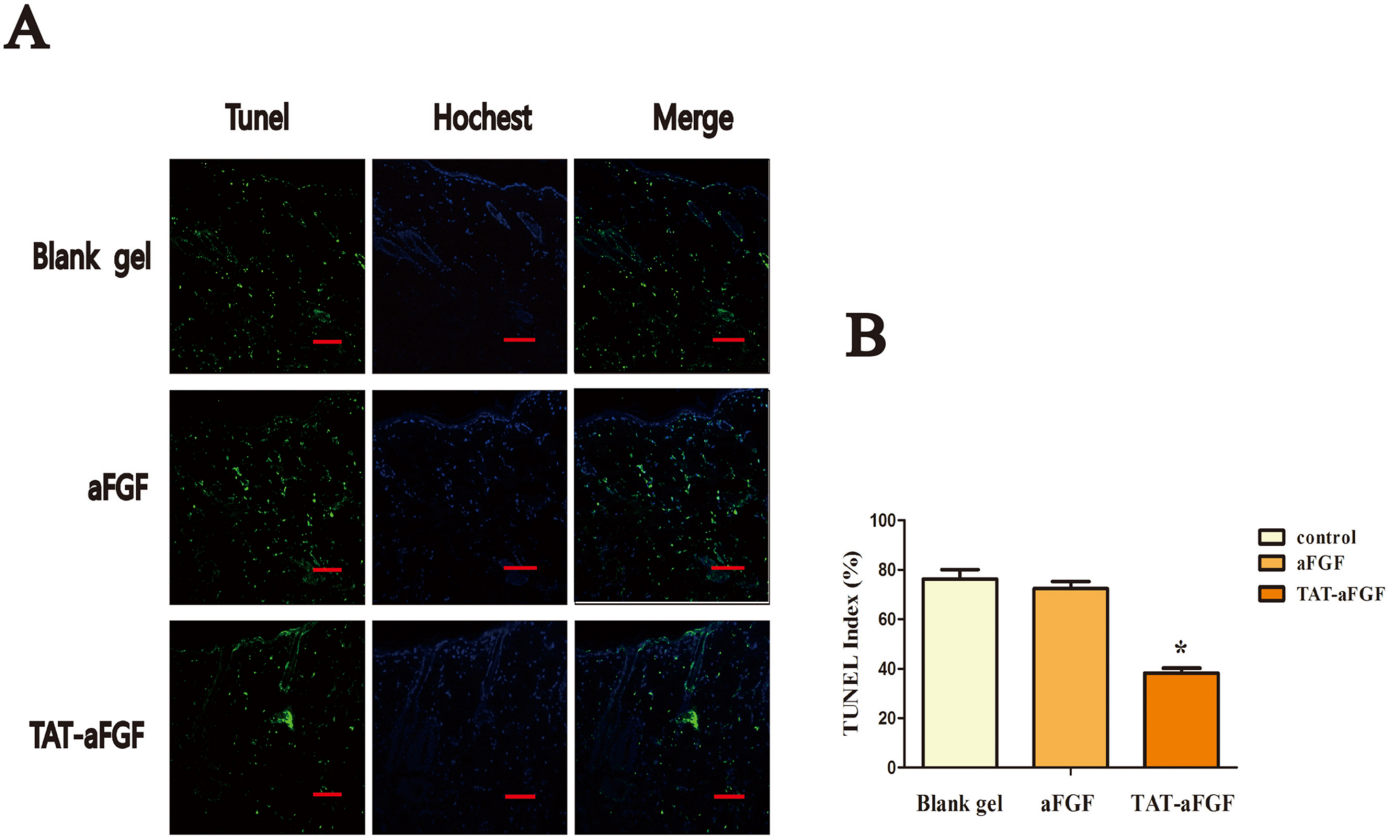
TUNEL assay of skins from deep tissue injury models. **A:** Terminal dUTP nick-end labeling (TUNEL, green) and DAPI (blue) staining was performed in TAT-aFGF, aFGF and control groups. Skin samples were harvested on day 0. Representative images are shown. Bar = 100 μm. **B:** TUNEL index of the three groups. * *p* < 0.05.

It has been shown that aFGF prevents the apoptosis of gut epithelial and myocardial cells that is triggered by ischemia-reperfusion injury [27, 28]. This effect contributes to the promotion of the ERK1/2 pathway and cell cycle progression as well as the maintenance of intracellular Ca^2+^ concentrations [28]. In our study, a TUNEL assay revealed that the index of apoptotic nuclear DNA breaks in ulcerated skin tissues (including epidermis, dermis and subcutaneous tissues), decreased significantly upon TAT-aFGF treatment (Fig 3A and B), suggesting that TAT-aFGF can efficiently penetrate the epidermal barrier and protect cells from apoptosis.

**Fig 4:**
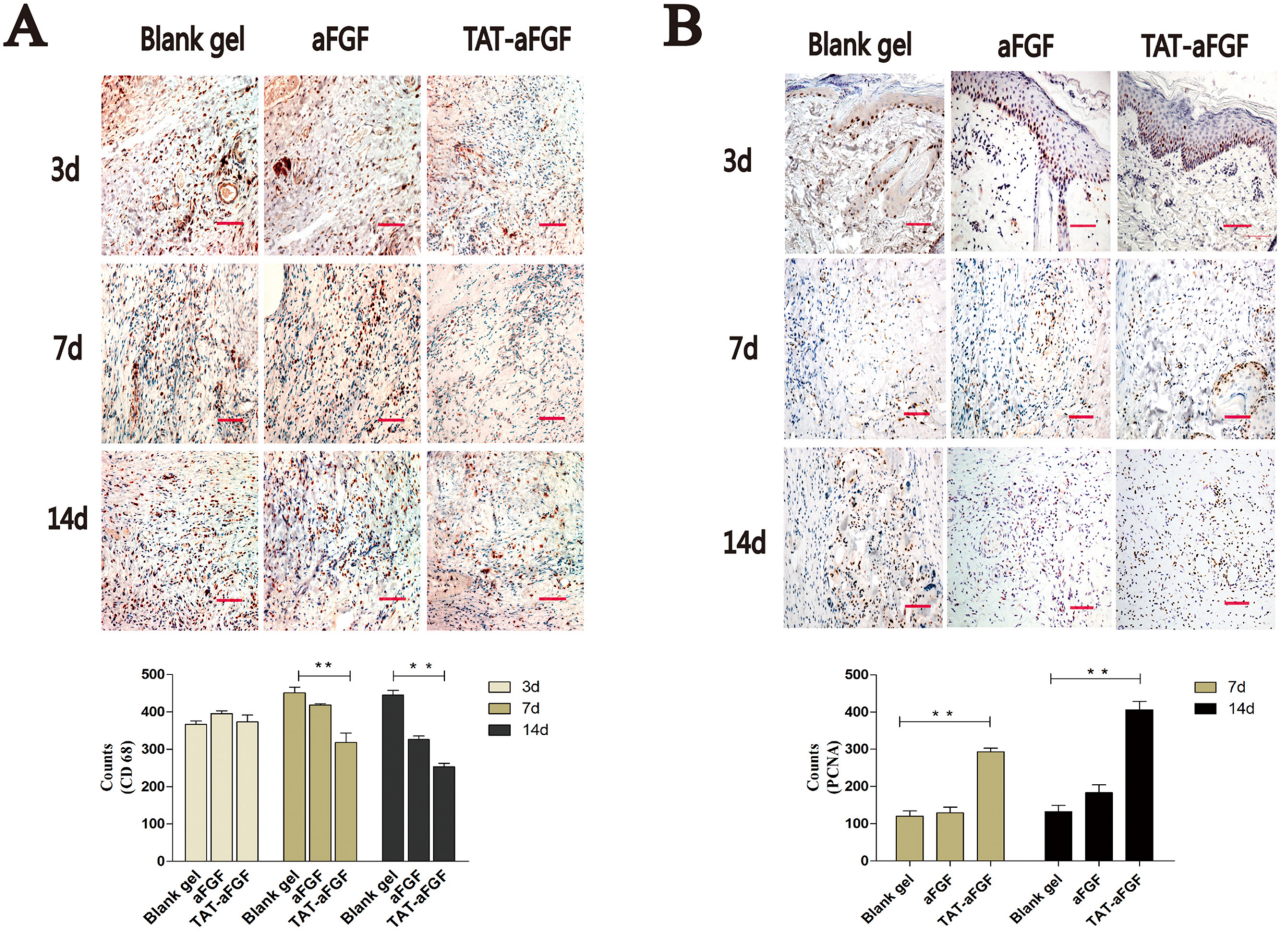
Effect of TAT-aFGF on CD68 and PCNA expression. **A:** The effect of TAT-aFGF, aFGF and control treatments on regional inflammation was assessed by immunohistochemical staining for CD68 (red). Representative images of the three groups on days 3, 7, 14 are shown. Bar = 100 μm. ** *p* < 0.01. **B:** On day 7 and 14, the PCNA expression (red) was detected in the three groups. Representative images were shown. Bar = 100 μm. ** *p* < 0.01.

Inflammation was evaluated in skins by staining for CD68 (specific for monocytes/macrophages). Before day 3, there was no significant difference in levels of CD68 between the three groups. However on day 7 and 14 the CD68 level in the TAT-aFGF group had decreased significantly compared with that of the CD68 level in the other two groups (both *p* < 0.01) (Fig 4A). Immunohistochemical analysis of PCNA showed that proliferation of epidermal cells, especially in the basal layer, was enhanced in the TAT-aFGF group compared to the control or aFGF groups. On days 7 and 14, the PCNA-positivity also remained higher in TAT-aFGF group compared to the other two groups (Fig 4B).

**Fig 5:**
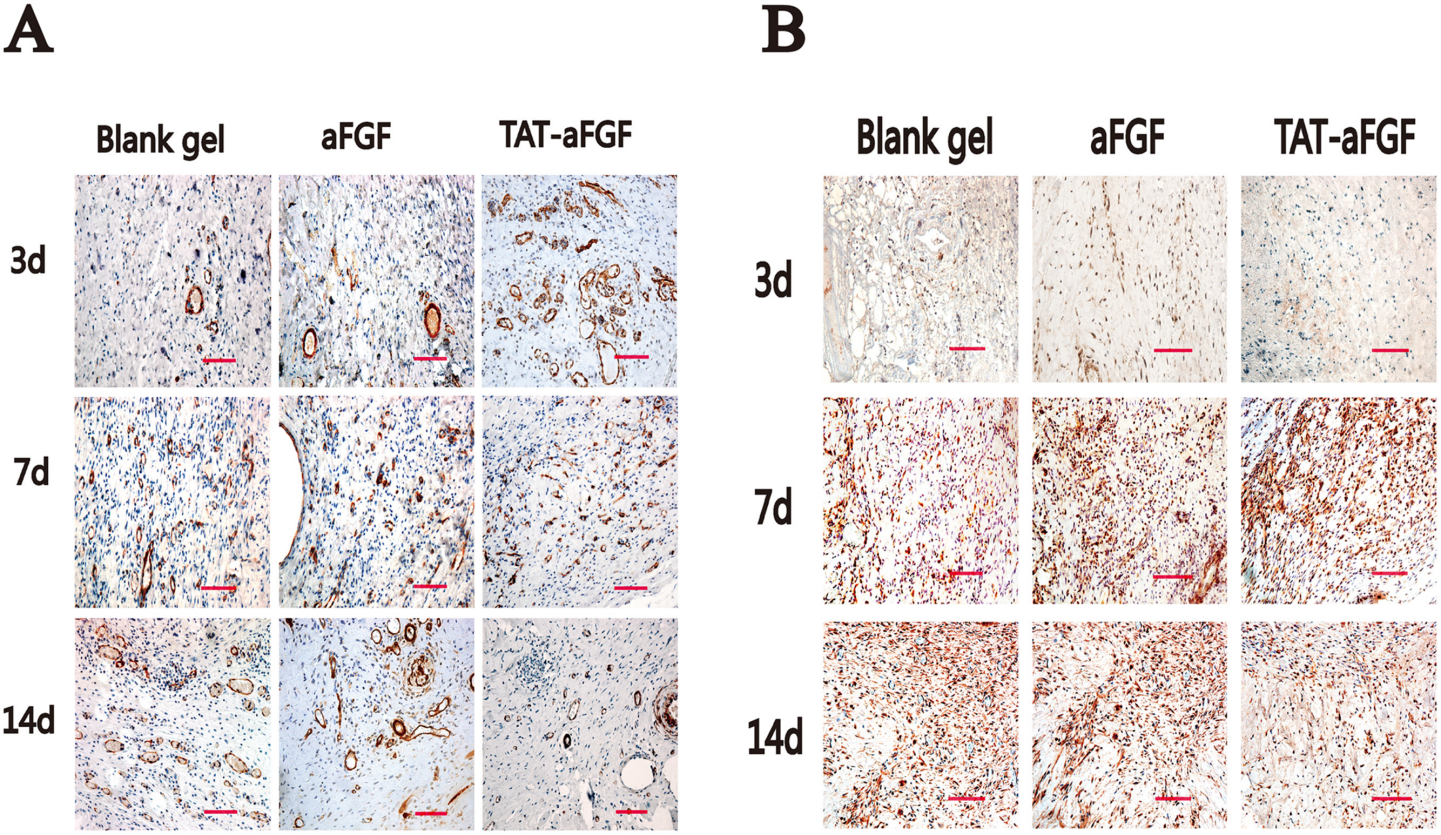
Effect of TAT-aFGF on TGF-β1 and α-SMA expression. **A**: On day 3, 7, and 14, the TGF-β1 expression was detected in TAT-aFGF, aFGF and control treatment groups. Bar = 100 μm. **B:** On day 3, 7, and 14, the α-SMA expression was detected in the three groups. Bar = 100 μm.

TGF-β1 induces α-SMA expression in subcutaneous fibroblasts, both *in vivo* and *in vitro* [29]. On day 3, there was no increase in TGF-β1 in the TAT-aFGF, aFGF or control groups. However, the TAT-aFGF group showed enhanced expression of TGF-β1 on day 7 followed by decreased expression on day 14, when compared with the control group (Fig 5A). No differences were found between the control and aFGF groups on day 7 or 14 (Fig 5A). Positive staining of α-SMA was observed underneath the pressure-loading area at the ulcer edge. Vascular smooth muscle cells were found in all groups at day 3 (Fig 5B). On day 7, myofibroblasts with higher α-SMA expression were observed in the connective tissues from the TAT-aFGF group (Fig 5B). However, on day 14, fewer α-SMA-positive cells were observed under the recovered neo-epidermis in the TAT-aFGF group compared with the control group (Fig 5B).

**Fig 6:**
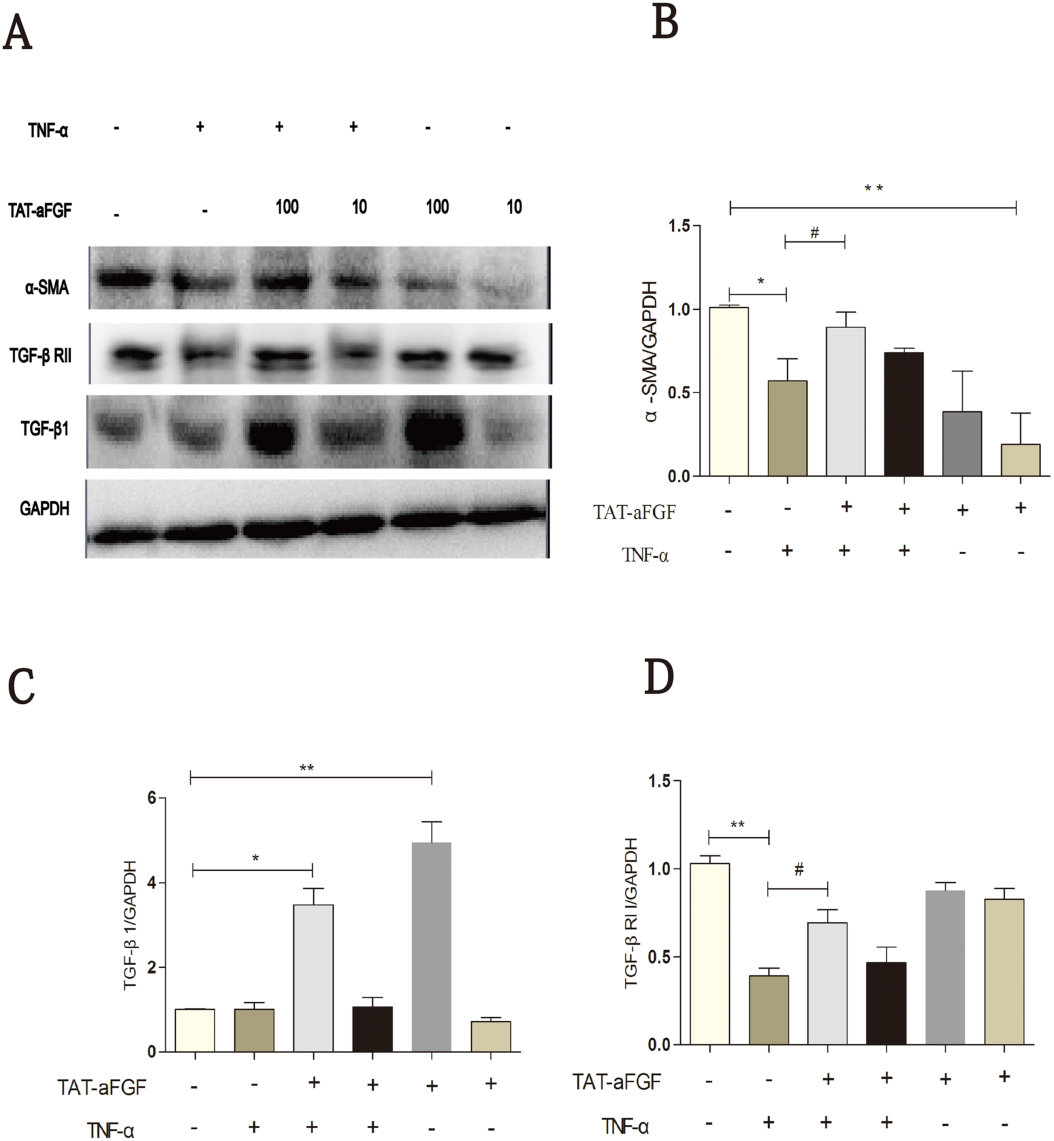
Western blot analysis of α-SMA, TGF-β1 and TGF-βRII in human dermal fibroblasts. **A:** Fibroblasts grown in a monolayer were serum-starved for 24 h before the stimulation of TNF-α (5 ng/ml) or TNF-α plus TAT-aFGF (10 ng/ml or 100 ng/ml). Total protein was extracted at the indicated time points. Western blotting was performed for α-SMA, TGF-β1, and TGF-βRII. **B, C, D:** α-SMA, TGF-β1, and TGF-βRII expression was normalized to GAPDH. Data were obtained from 3independent experiments. * *p* < 0.05, ** *p* < 0.01.

Western blotting revealed that TNF-α treatment decreased α-SMA expression in human dermal fibroblasts (Fig 4), consistent with data from a previous study [30]. Application of TAT-aFGF reversed the inhibitory effect of TNF-α on α-SMA expression; however, TAT-aFGF alone also decreased α-SMA expression (Fig 4B). This effect contributed partially to the changes in the levels of TGF-β1 and TGF-βRII (Fig 4A). In the presence of TNF-α, TAT-aFGF (100 ng/ml) restored TGF-β1 and TGF-βRII expression in human dermal fibroblasts (Fig 6C and 6D).

## Discussion

In this study, we found that TAT facilitated the delivery of aFGF across the cutaneous barrier and the accumulation aFGF in the dermis or subcutaneous tissues. TAT-aFGF enhanced the healing process of deep tissue injury under the skin mediated by aFGF in a rat model. Moreover, TAT-aFGF had higher potential to penetrate the membranes of human dermal fibroblasts *in vitro*, compared with the aFGF alone. In addition, TAT-aFGF reversed the suppressive effect of TNF-α on α-SMA expression and restored TGF-β1 and TGF-βRII expression in dermal fibroblasts. Therefore, our results demonstrate that TAT-aFGF has a favorable therapeutic effect on the healing of deep tissue injury under the skin.

Consistent with a previous report that TAT-aFGF enhance the accumulation of aFGF in the retina following its topical administration to the ocular surface [17], our results show that topical application of TAT-aFGF also enhances aFGF accumulation in both dermis and subcutaneous tissues. In areas of wounded skin, the number of apoptotic cells was significantly reduced with TAT-aFGF treatment, when compared with aFGF or control treatment on day 0 as indicated by the decreased TUNEL index. This finding mirrored the higher penetration efficiency of TAT-aFGF protein in transdermal delivery experiments. In a previous study, we demonstrated that TAT-aFGF-His treatment can reduce cell apoptosis in ischemia–reperfusion rats [17]. Hence, we speculate here that aFGF accumulation in cutaneous tissue ameliorates fibroblast apoptosis. The possible mechanisms underlying such a phenomenon deserve further studies e.g. the effect of aFGF on the activation of FGF receptors in skin tissues.

PCNA is a marker of cell proliferation [31]. We found that PCNA expression was strongly detected in cells within all tissue layers of skin in the TAT-aFGF group. The TAT-aFGF group also showed reduced apoptosis of cells under the epidermis. Hence, the enhanced proliferation and reduced apoptosis of cells which are the main components of skin (such as keratinocytes) could be contributing to the accelerated improvement on pressure ulcer healing, resulting in an early separation of eschar from the skin. Furthermore, enhanced deposition and more complete organization of collagen fibers were observed in the TAT-aFGF group, while the collagen fibers were often irregularly arranged in both the aFGF and control groups. In addition, the contracture of connective tissue was more severe in the control than TAT-aFGF group. These results indicate that TAT-aFGF application leads to better recovery of injured cutaneous tissues and amelioration of chronic scar formation, which was also observed with basic FGF in *vivo* [32].

During wound repair and skin regeneration, myofibroblasts are a specialized subgroup of cells with the features of both fibroblasts and smooth muscle cells [22], the latter of which are characterized by the expression of α-SMA. It has been widely accepted that myofibroblasts numbers correlate not only with wound closure but also with remodeling of the ECM [24]. TNF-α, secreted by inflammatory cells, inhibits ECM synthesis while activating matrix metalloproteinases [33]. A recent study showed that TNF-α suppressed TGF-β1-induced α-SMA expression in human dermal fibroblasts and, furthermore, that JNK phosphorylation and TGF-βRII activation were involved in this process [30, 34]. Although TAT-aFGF showed no detectable effect on the early phase of inflammation in rat skins, α-SMA expression was already significantly higher in this group compared with aFGF alone or controls. Interestingly, α-SMA levels were lower in the TAT-aFGF group than the other two groups on day 14, consistent with a decreased inflammation status in the TAT-aFGF group. Such regulation of α-SMA synthesis by TAT-aFGF not only favors the healing of wounds, but also reduces the formation of scars. These findings further support the opinion that TAT-mediated aFGF delivery efficiently promotes tissue wound remodeling and normally functioning tissue recovery [35]. Finally, our results demonstrate, for the first time, that the upregulation of both TGF-β1 and TGF-βRII is involved in mediating the effect of TAT on aFGF delivery.

## Conclusion

In summary, our results demonstrate that fusion with TAT enhances the penetration of aFGF through the epidermis and facilitates the healing of DTI in skin. This feature of TAT may be related to its regulation of α-SMA expression and restoration of TGF-β1 and TGF-βRII synthesis by human dermal fibroblasts. Application of TAT-aFGF can be used to developed more efficient and less invasive approaches for the treatment of DTI, such as ulcers resulting from physical damage or the diabetic condition.
